# Rationale behind the European Thyroid Association 2024 Guideline to treat the Allan–Herndon–Dudley syndrome with tiratricol?

**DOI:** 10.1530/ETJ-25-0153

**Published:** 2025-10-24

**Authors:** Jan Olof G Karlsson

**Affiliations:** Department of Biomedical and Clinical Sciences, Division of Clinical Chemistry and Pharmacology, Linköping University, Linköping, Sweden

In 2024, the European Thyroid Association published its guidelines on the diagnosis and management of genetic disorders of thyroid hormone transport, metabolism and action, with special reference to monocarboxylate transporter 8 (MCT8) deficiency, published in the European Thyroid Journal (1). I presume that these guidelines were crucial for the European Union’s decision to approve tiratricol (Emcitate®), under an exclusive marketing authorization label. However, the rationale behind these guidelines is in my view questionable.

MCT8 deficiency, also known as Allan–Herndon–Dudley syndrome (AHDS), is an ultra-rare genetic defect characterized by i) chronic thyrotoxicosis, caused by peripheral hyperthyroidism, and ii) severe neuromotor and neurocognitive disability, caused by cerebral hypothyroidism (1, 2, 3).

Tiratricol, also known as TRIAC or triiodothyroacetic acid, is a thyroxine metabolite, which has been studied since the 1950s and has for a long period of time been known to relieve peripheral thyrotoxicosis (4). Egetis Therapeutics AB is in the process of commercializing tiratricol as an AHDS remedy. The company has received orphan drug designation (ODD) in both the EU and the USA for treating this syndrome. Tiratricol has demonstrated clinical efficacy on the thyrotoxicosis part of the syndrome, by normalizing serum triiodothyronine levels (2, 3).

Importantly though, no treatment, including tiratricol, has been shown to improve neuromotor and neurocognitive development in children with MCT8 deficiency (1). Accordingly, Egetis reported in a press release on June 19, 2024, that its phase II study (NCT02396459) failed to improve neuromotor and neurocognitive development. The company was, nevertheless, confident that the already shown efficacy on peripheral thyrotoxicosis (3) should be enough for gaining marketing authorization and exclusive rights in both Europe and the USA, as communicated in the press release.

Without any proof that tiratricol improves the neuromotor and neurocognitive part of AHDS (1), it is in my opinion relevant to provocatively question if the 2022 European Thyroid Association Guideline for the management of severe cases of Graves’ disease in pediatric patients, aiming for complete thyroidectomy and life-long administration of optimally titrated levothyroxine, is superior to life-long treatment with tiratricol. In contrast to levothyroxine, experience from life-long treatment is lacking, which, from several aspects, is advantageous for the former treatment.

Taking into consideration that MCT8 deficiency is an ultra-rare condition, the cost of treating each patient with tiratricol under an exclusive marketing authorization label is expected to be extremely high in comparison with that of the conventional method, as applied in severe cases of Graves’ disease. In my view, the so far established methods to treat the peripheral thyrotoxicosis part of MCT8 deficiency, in line with the 2022 European Thyroid Association Guideline for the management of severe cases of Graves’ disease in pediatric patients, are superior to those of tiratricol (5).

## Declaration of interest

JOGK is a founder and a former employee of PledPharma (now Egetis Therapeutics AB). He is the first inventor on three granted patent families (e.g. US8377969, US8633174, US9187509 and US10688087) covering the therapeutic use of calmangafodipir [Ca_4_Mn(DPDP)_5_], which are owned by PledPharma/Egetis. JOGK is, furthermore, an inventor on a granted patent family (e.g. US10888553) covering the therapeutic use of fodipir (DPDP) to treat platinum-induced peripheral sensory neuropathy, which is owned by Karlsson-Tuner Invest AS.

## Funding

This work did not receive any specific grant from any funding agency in the public, commercial or not-for-profit sector.
